# Chiral sensitivity of medetomidine lateral flow immunoassay test strips

**DOI:** 10.1186/s12954-025-01387-6

**Published:** 2026-01-02

**Authors:** Anita Amate, Marya Lieberman

**Affiliations:** Department of Chemistry and Biochemistry, University of Notre Dame, Notre Dame, IN 46556 USA

## Abstract

Medetomidine has recently emerged in the illicit drug supply in the United States, and lateral flow immunoassay test strips are an inexpensive and easy-to-use field screening option for detecting this highly potent sedative. However, the drug is chiral, and the response of test strips to the different enantiomers has not been reported. This study evaluated the chiral sensitivity of 7 lots of medetomidine test strips produced by two manufacturers. Test strips were assessed using solutions of dexmedetomidine, levomedetomidine, and racemic medetomidine at varying concentrations, water types, and temperature conditions. Specificity was evaluated by testing structurally related compounds and other interferences. Multiple lots of medetomidine strips responded only to dexmedetomidine. These strips detect racemic medetomidine but give a negative result for pure levomedetomidine. Other lots of medetomidine strips required both dexmedetomidine and levomedetomidine to be present to give a positive result–either pure dexmedetomidine or pure levomedetomidine gave negative results. All strips performed best in 18 MΩ water. at temperatures of 25 °C or below. Xylazine did not cross-react. The veterinary sedative detomidine HCl caused false positives on all the strips at concentrations down to 0.07 mg/mL, and levamisole gave faint test lines (which can be misread as false positives) for almost all the strips at 2 mg/mL. Organizations selecting test strips for public health applications should select strips that can detect both dexmedetomidine and racemic versions of this sedative.

**Supplementary Information:**

The online version contains supplementary material available at 10.1186/s12954-025-01387-6.

## Introduction

In the US, drug-related overdoses and deaths rapidly increased from 2000 to 2024, with synthetic opioids like fentanyl being the primary drivers of this crisis. Recently, the situation has been further complicated by the emergence of veterinary sedatives into the illicit drug supply. The centers for disease control and prevention (CDC) has been tracking the growing presence of animal sedatives such as xylazine and medetomidine, which are increasingly detected alongside fentanyl [1]. These sedatives, often added without the knowledge of people who use drugs, increase the risks associated with overdose and complicate medical and public health responses. Medetomidine is an α2 adrenergic receptor agonist that is currently not regulated as a controlled substance under national drug schedules (e.g., DEA schedules in the U.S.). It induces effects such as bradycardia and reduced cardiac output by stimulating peripheral and central α2 receptors.

Medetomidine has emerged as a notable adulterant in the illicit opioid supply in parts of the United States. First identified by the U.S. Drug Enforcement Administration’s National Forensic Laboratory Information System (NFLIS) in 2021, it has since been found in street drugs with fentanyl analogs, xylazine, heroin, and cocaine. Since mid-2022, multiple overdose cases involving medetomidine have been reported in Ohio, Florida, and Canada [2]. In mid-to-late 2023, medetomidine started to appear in toxicology specimens of patients admitted to emergency departments after suspected opioid overdoses in Missouri, Colorado, Pennsylvania, California, and Maryland [1]. In 2024, medetomidine was found in the recreational drug supply and biological specimens from individuals using illicit opioids in at least 18 U.S. states [3, 4]. The clinical evidence indicates that medetomidine, increasingly found as an adulterant in illegally manufactured opioids, can cause severe withdrawal symptoms when discontinued after prolonged exposure [5]. In a report of 23 patients in Pittsburgh (October 2024–March 2025), individuals exhibited profound autonomic hyperactivity, including hypertension, tachycardia, and agitation, nausea, vomiting, often requiring intensive care and dexmedetomidine infusion for stabilization [6]. These findings highlight that medetomidine exposure carries risks beyond overdose, with withdrawal that can be difficult to manage and potentially dangerous, even when standard opioid treatments are provided [6].

**Fig. 1:**
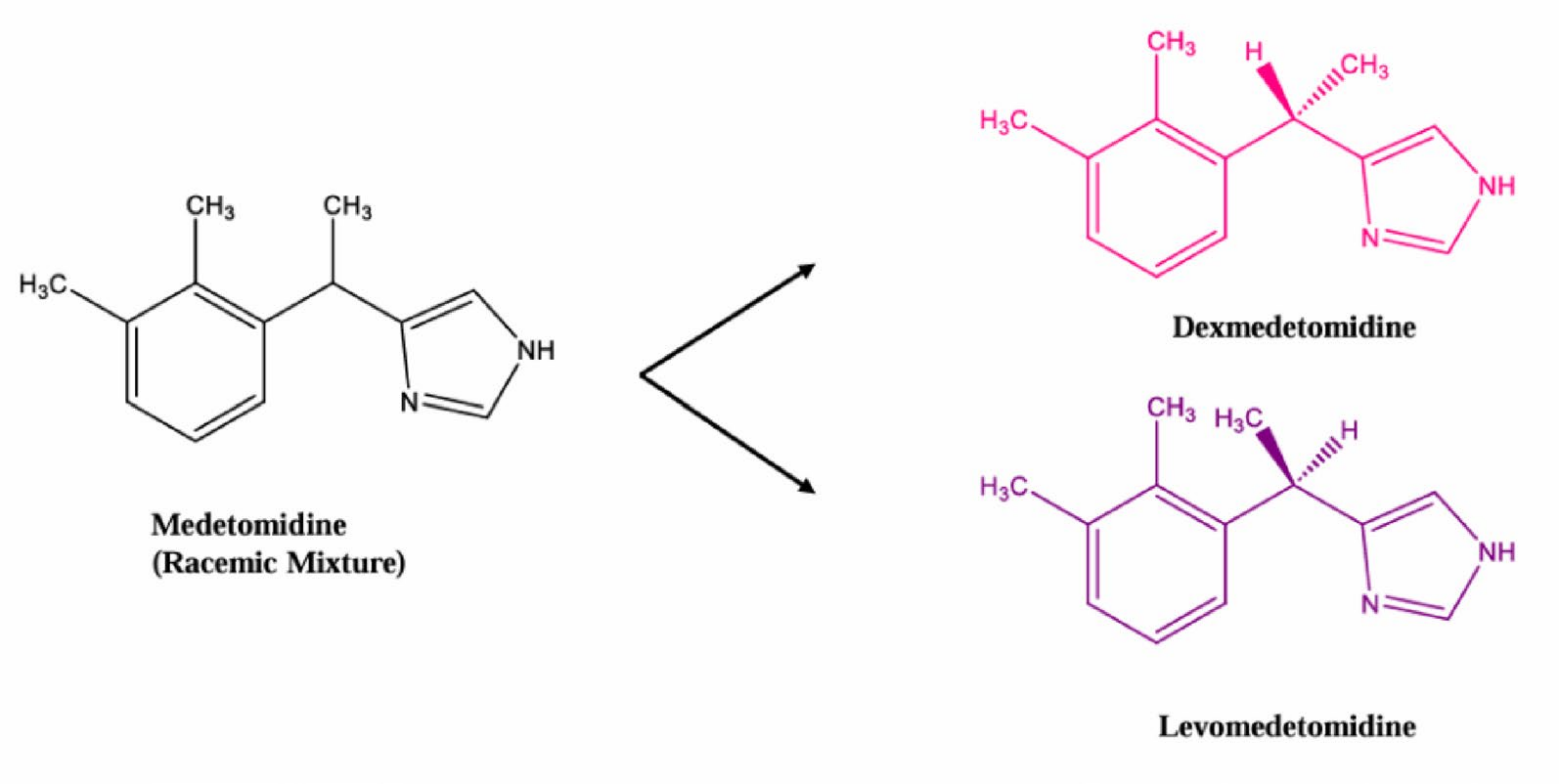
Chemical structures of medetomidine optical isomer

Medetomidine is a racemic mixture of two optical isomers (Fig. 1). All the activity is due to the dextrorotary isomer, dexmedetomidine; the levorotatory isomer is pharmacologically inactive [7–9]. The racemic mixture (1:1 dex: levo) is used in veterinary medicine but is not approved for use in humans. Racemic medetomidine is 200 to 300 times more potent than xylazine, with a longer duration of sedation and analgesia [10]. Dexmedetomidine is a Food and Drug Administration–approved drug for humans; it is used for sedation in intensive care units, with careful monitoring to avoid side effects [11]. Medetomidine in street drugs could originate from diversion of veterinary (racemic medetomidine) or human (dexmedetomidine) medications, or from illegally synthesized medetomidine, which in turn could be either racemic or homochiral. Exposure to racemic or dexmedetomidine can lead to central nervous system depression and sedation, contributing to increased morbidity and mortality among people who use drugs. Chirality is critical for the pharmacology and toxicity of many drugs. Enantiomers can have different pharmacokinetic and pharmacological profiles because the human body is a chiral environment [12]. For example, in the US, d-methamphetamine is an active stimulant that is a controlled substance, while l-methamphetamine is much less active [13]. Other examples include amphetamine, methadone, MDMA, and medetomidine, where the active effects are primarily associated with one enantiomer.

Understanding how to identify and respond to medetomidine exposure in the context of illicit drug use is crucial. Some research studies suggest that atipamezole may help reverse the effects of medetomidine [14, 15] however, currently, there are no FDA-approved or emergency medical system-approved treatments to counteract the α2-adrenergic receptor (α2-AR) depressant effects of medetomidine, so avoiding it is the safest option. Medetomidine test strips are a simple, powerful technology for the detection of this sedative in the drug supply, providing a practical way for individuals to stay safer.

Lateral flow immunoassay test strips depend on antibodies to bind their targets [16]. Antibodies are inherently chiral, so they often have very different binding constants for different optical isomers of a target molecule. Got and Scherrmann review strategies and considerations for the generation of antibodies targeting chiral drugs [17]. Haptens such as the one shown in Fig. S3 are commercially available (e.g., AAT Bioquest); this medetomidine hapten exposes the chiral carbon center to the immune system for the generation of antibodies. A test strip manufacturer might select monoclonal antibodies that bind dexmedetomidine, mix one monoclonal antibody for dexmedetomidine and one monoclonal antibody for levomedetomidine, or could use polyclonal antibodies that recognize both dex and levo medetomidine. To determine the chiral recognition strategies in use in commercial medetomidine strips, we tested multiple lots of medetomidine and dexmedetomidine test strips at various ratios of dexmedetomidine and levomedetomidine. We also evaluated the limit of detection (LOD) for racemic medetomidine and dexmedetomidine in various water types and temperature conditions, and evaluated interferences caused by other common components in the illicit drug supply and by chemicals with similar structures to medetomidine.

## Materials and methods

Five lots [DOAB24080502, DOAB24100900, DOAB24100902, DOAB25060002, DOAB25040024] of Rapid Response Medetomidine test strips were received from BTNX Inc. (Pickering, ON, Canada, New Buffalo); BTNX informed us that the strips with lot numbers DOAB24080502 and DOAB24100900 were early versions, while the strips with lot numbers DOAB24100902, DOAB25060002, and DOAB25040024 DOAB25040024 were redesigned to specifically target dexmedetomidine and are thus called ‘dexmedetomidine’ strips. They were manufactured by BTNX.

Two lots [MTM25050002, MTM24100902] of WiseBatch-branded Medetomidine test strips were received from the WiseBatch Harm Reduction Group (791 Leeward Way, Costa Mesa, CA); these strips are manufactured by W. H. P. M (Irwindale, CA) (Table 1, Figs. S1a, S1b). Test strips are intended for forensic use only and are specifically designed for analyzing solutions prepared from solid drug forms.

**Table 1:** Test strips used in this study

Test strip type	Brand	Lot number	Expiry date	Stated cutoff
Medetomidine	BTNX	DOAB24080502	2026/08/07	1000 ng/ml
Medetomidine	BTNX	DOAB24100900	2027/10/18	1000 ng/ml
Medetomidine	WiseBatch	MTM25050002	2028/05/27	Not Given
Medetomidine	WiseBatch	MTM24120002	2026/12/17	Not Given
Dexmedetomidine	BTNX	DOAB24100902	2027/10/18	1000 ng/ml
Dexmedetomidine	BTNX	DOAB25060002	2028-06-11	1000 ng/ml
Dexmedetomidine	BTNX	DOAB25040024	2028-04-24	1000 ng/ml

Medetomidine hydrochloride [Item #38582purity > 98%, crystalline solid], dexmedetomidine hydrochloride [Item #15,581 purity- ≥ 99%, solid], and levomedetomidine hydrochloride [Item #35,201, purity- ≥ 98%, solid] were purchased from Cayman Chemical Company (Ann Arbor, MI, USA). For the 5 mg fill sizes used, the supplier reports a tolerance of approximately – 2% to + 5%, meaning the actual content may range from about 4.9 mg to 5.25 mg. Other materials for interference testing (Table 2) were obtained from either Cayman Chemical or Sigma Aldrich (St. Louis, MO, USA). 18 MΩ water was used to prepare all standards and samples; this highly purified water is comparable to deionized or DI water. The tap water used in this study comes from the University of Notre Dame’s water system; it is extremely hard, with over 500 mg/L of total dissolved solids including calcium, sulfate, magnesium, chloride, and iron [18, 19].

**Table 2:**
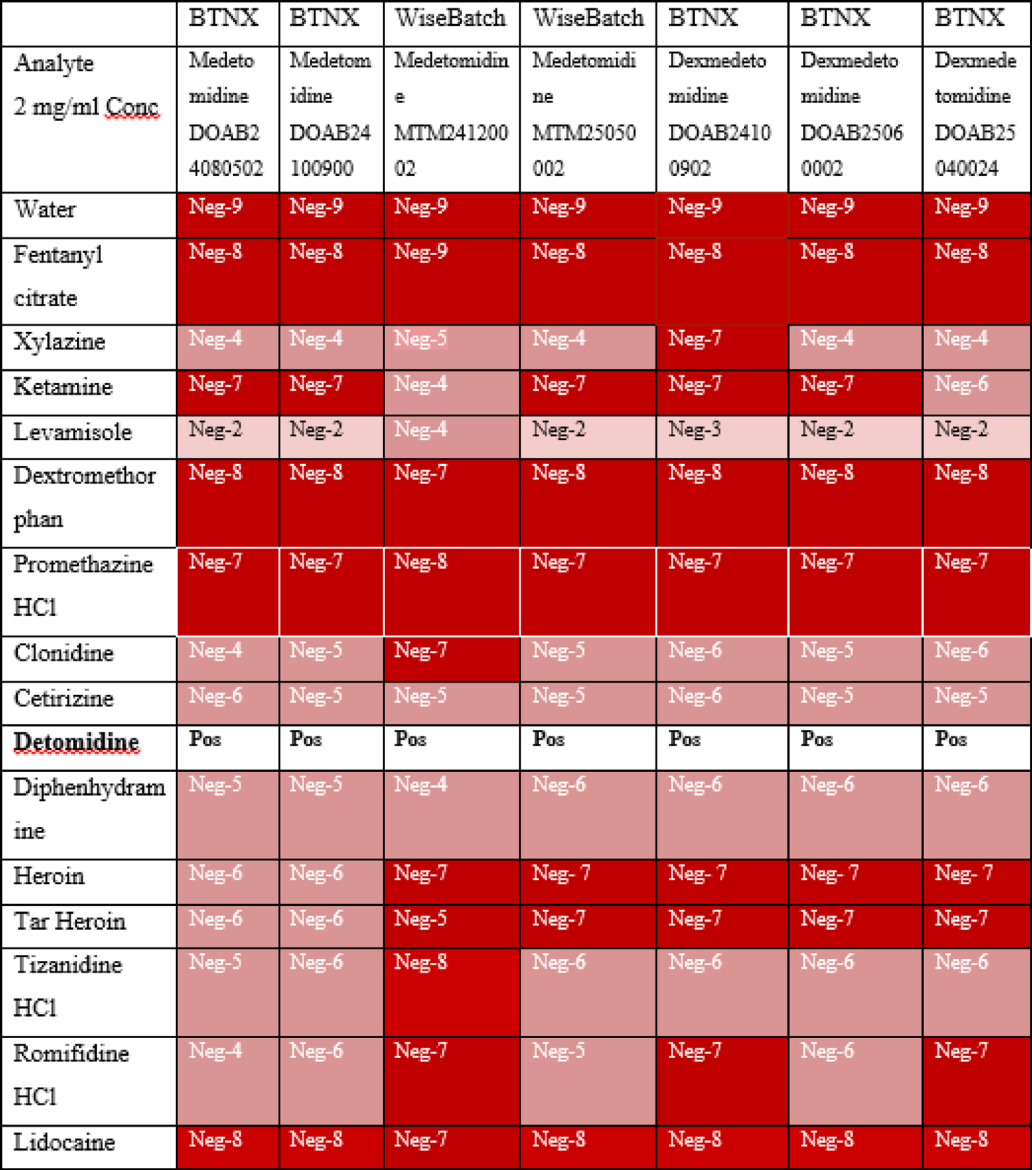
Performance of test strips with potential interferences

A negative result produces a red color at the test line; the intensities of these colors based on the intensity score card are indicated numerically and also coded as the intensity of red color in each cell. A strong red color (Neg-4 or greater) indicates a clear negative result, while a weak pink color (Neg-1, Neg-2, or Neg-3) indicates a faint test line that might be misread as a false positive

### Test strip analysis

In this study, we evaluated the performance of medetomidine rapid test strips [BTNX, WiseBatch] that target medetomidine in street drugs. All the test strips were kept in their sealed foil pouches at room temperature until they were used. The test strips were dipped into the test solution for 15 s and then placed on a clean, dry surface for 5 min. The test strips were first evaluated by eye to check for inconclusive results, such as missing control lines (none were observed), and to read the strip result. Following the manufacturer’s directions, any visible test line, no matter how faint, was recorded as a negative result. The intensity of the test lines was recorded using a 1–10 visual intensity scorecard (lateralDX, #12,082,021,www.lateral.com [20]. Photographs of test strips were captured using an iPad 10 in the laboratory lighting.

### Interference

Approximately twenty milligrams (mg) of each interfering substance (Table 2) were weighed using an analytical balance and transferred into a labeled vial. Each sample was dissolved in 10 ml (mL) of 18 MΩ water to achieve a final concentration of 2 mg/mL. A single test strip from each lot was used to test each solution.

### Limit of detection (LOD)

A 5 mg bottle of racemic medetomidine hydrochloridewas dissolved in 5 mL of 18 MΩ water, giving a stock solution with a final concentration of 1 mg/mL. Serial dilutions were prepared from this stock solution to obtain final concentrations ranging from 500 ng/mL to 5000 ng/mL in 18 MΩ water. If needed, these concentrations may be converted to the concentrations of medetomidine free base by multiplying by 0.844 (the ratio of the formula weights).

A 5 mg bottle of dexmedetomidine hydrochloride was dissolved in 5 mL of 18 MΩ water, preparing a stock solution with a final concentration of 1 mg/mL. Serial dilutions were prepared from this stock solution to obtain final concentrations ranging from 500 ng/mL to 5000 ng/mL in 18 MΩ water. If needed, these concentrations may be converted to the concentrations of dexmedetomidine free base by multiplying by 0.844 (the ratio of the formula weights).

Medetomidine and dexmedetomidine test strips were evaluated at each concentration in triplicate at 25 °C.

### Variation of the LOD under different environmental conditions

#### Tap water

Medetomidine and dexmedetomidine hydrochloride solutions of 10,000 ng/mL were prepared in tap water using the 1 mg/mL stock solutions, giving a 99% tap water solution. The stock solutions were further diluted to obtain concentrations ranging from 500 to 5000 ng/mL in tap water. Medetomidine and dexmedetomidine test strips were evaluated at each concentration in triplicate at 25 °C.

#### LOD variations with temperature

To conduct the testing at a lower temperature, the solutions and test strips were stored in a cold room at 5 °C for 2 h before testing. The testing was carried out inside the cold room. A similar experiment was conducted at 38 ℃. The solutions and test strips were placed in an incubator for 2 h to equilibrate. Then, the strips were added to the test solutions and allowed to run for 15 s. Afterward, they were laid flat inside the incubator for 3 min before reading the test results.

#### Dex: levo threshold study

Solutions of dexmedetomidine and levomedetomidine were prepared in 18 MΩ water. at concentrations ranging from 5000 ng/mL to 500 ng/mL. The solutions were then mixed with varying concentrations. Testing ratios were set such that dexmedetomidine was maintained at a constant concentration of 5000 ng/mL, while levomedetomidine varied from 3000 ng/mL to 500 ng/mL, and vice versa. That test result is shown in Fig. 4, and pictures of test strips are provided in the supporting information. (SI Table 4).

## Results and discussion

### Limits of detection for medetomidine and dexmedetomidine

**Fig. 2:**
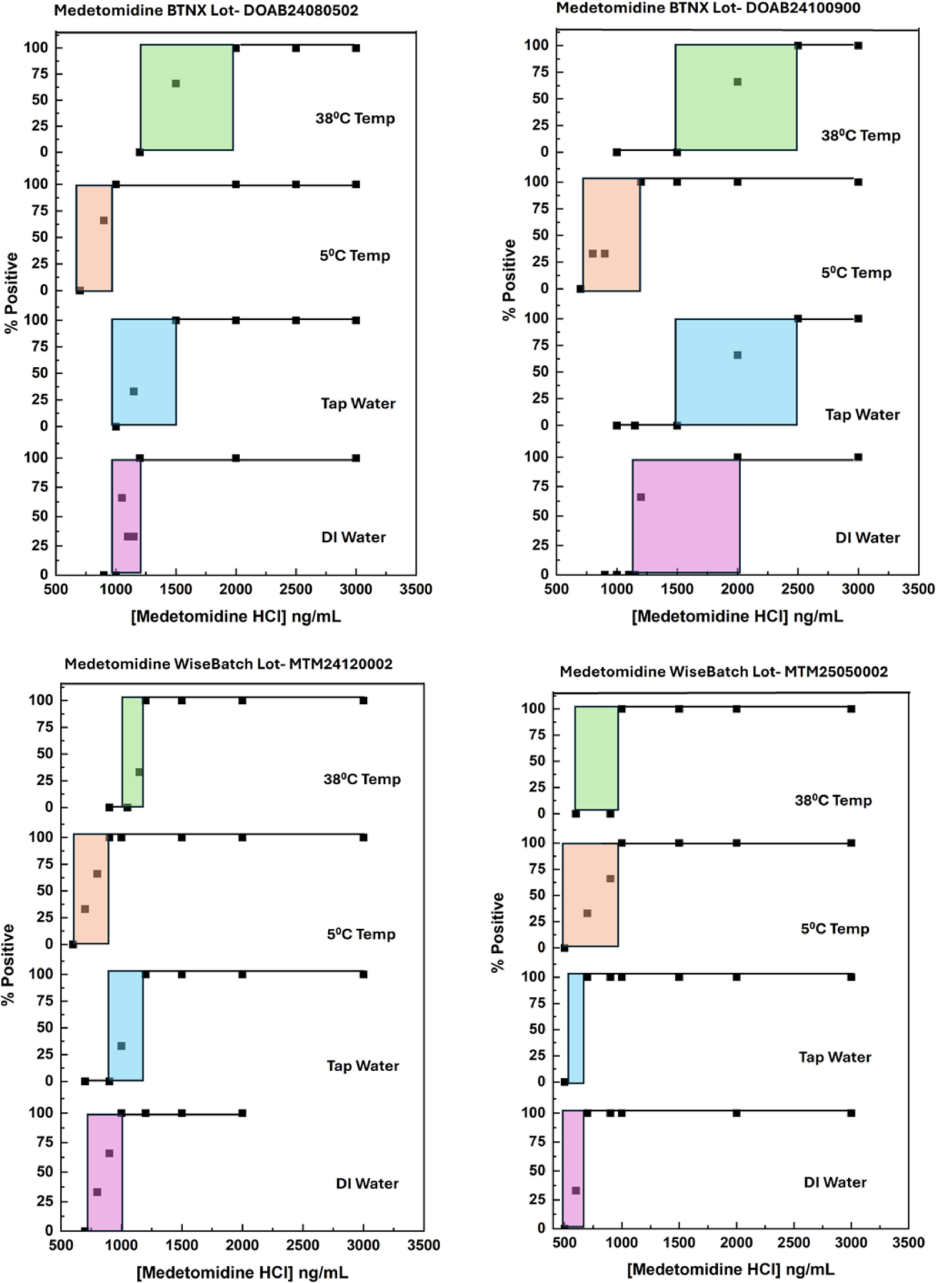
Sensitivity curves for medetomidine hydrochloride observed under various conditions. Shaded areas show inconclusive ranges where both positive and negative results were obtained on different strips in the triplicate testing

**Fig. 3:**
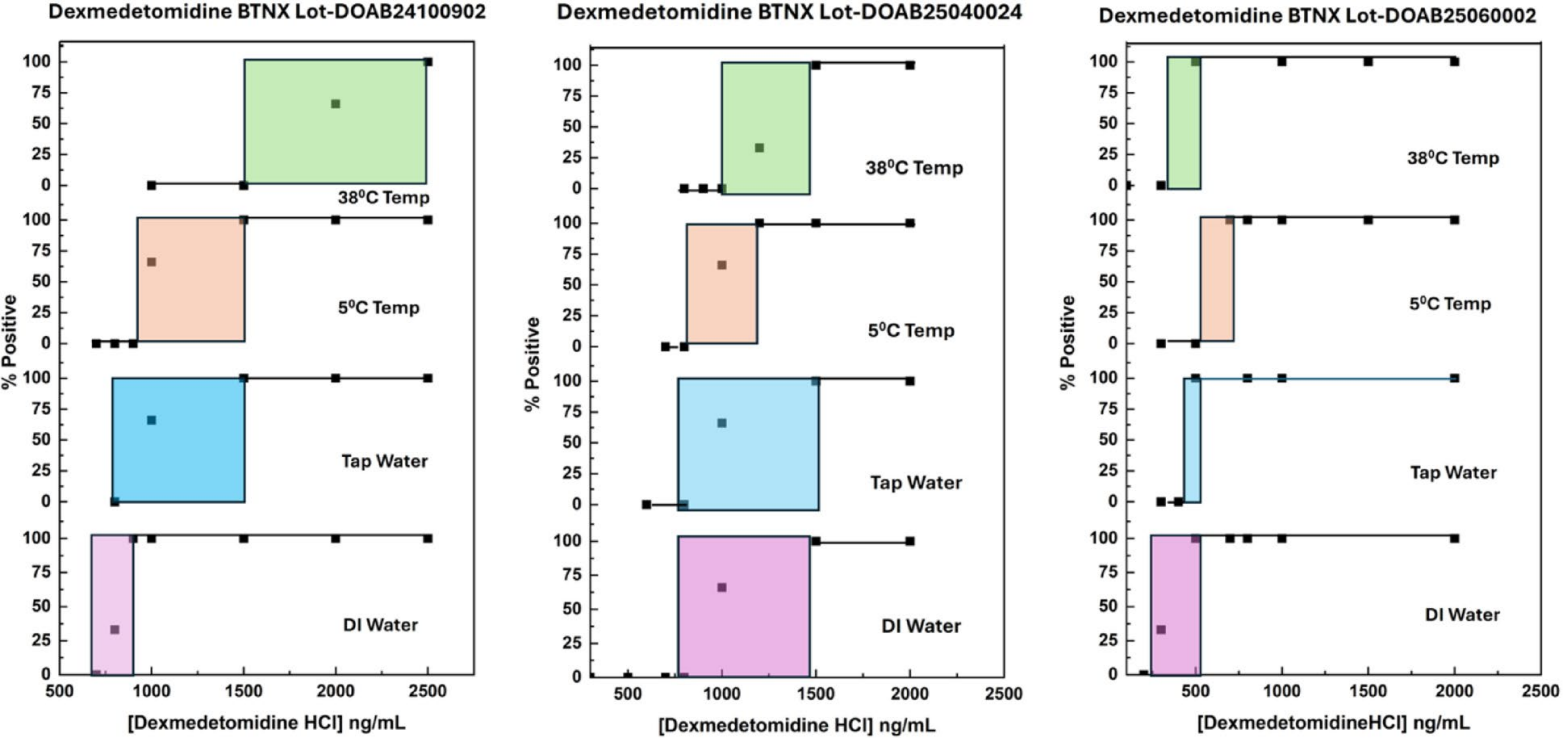
Sensitivity curves for dexmedetomidine hydrochloride observed under various conditions. Shaded areas show inconclusive ranges where both positive and negative results were obtained on different strips in the triplicate testing

The limits of detection of four lots of medetomidine test strips and three lots of dexmedetomidine test strips for their targets ranged from 500 to 2000 ng/mL in 18 MΩ water. Figure 2 shows results for the detection of racemic medetomidine, and Fig. 3 shows results for the detection of dexmedetomidine. The limit of detection (LOD) was defined as the lowest concentration of the target at which three replicate tests produced positive results. The limit of exclusion (LOE) was defined as the highest concentration for which three replicate tests produced consistent negative results. An inconclusive results range, represented by the colored boxes in the panels of Figs. 2 and 3, lies between the LOD and LOE.

The LOD values were better (i.e., had lower values) in 18 MΩ water. and at low temperatures (25 °C or 5 °C) than in tap water and at high temperatures (38 °C). These observations are consistent with recent studies examining the performance of fentanyl test strips in various environmental conditions, which found that elevated temperatures reduced the ability of the target to bind to the antibodies on the test line [21]. Fluctuations in pH and ionic strength across different water matrices are known to affect lateral flow immunoassay performance by modulating the electrostatic and non-covalent forces that govern antibody–antigen binding [22, 23]. Our local tap water is extremely hard, with calcium and magnesium concentrations near the limit of what the US EPA considers potable.

### Dex: levo ratio effects on medetomidine detection thresholds

**Fig. 4:**
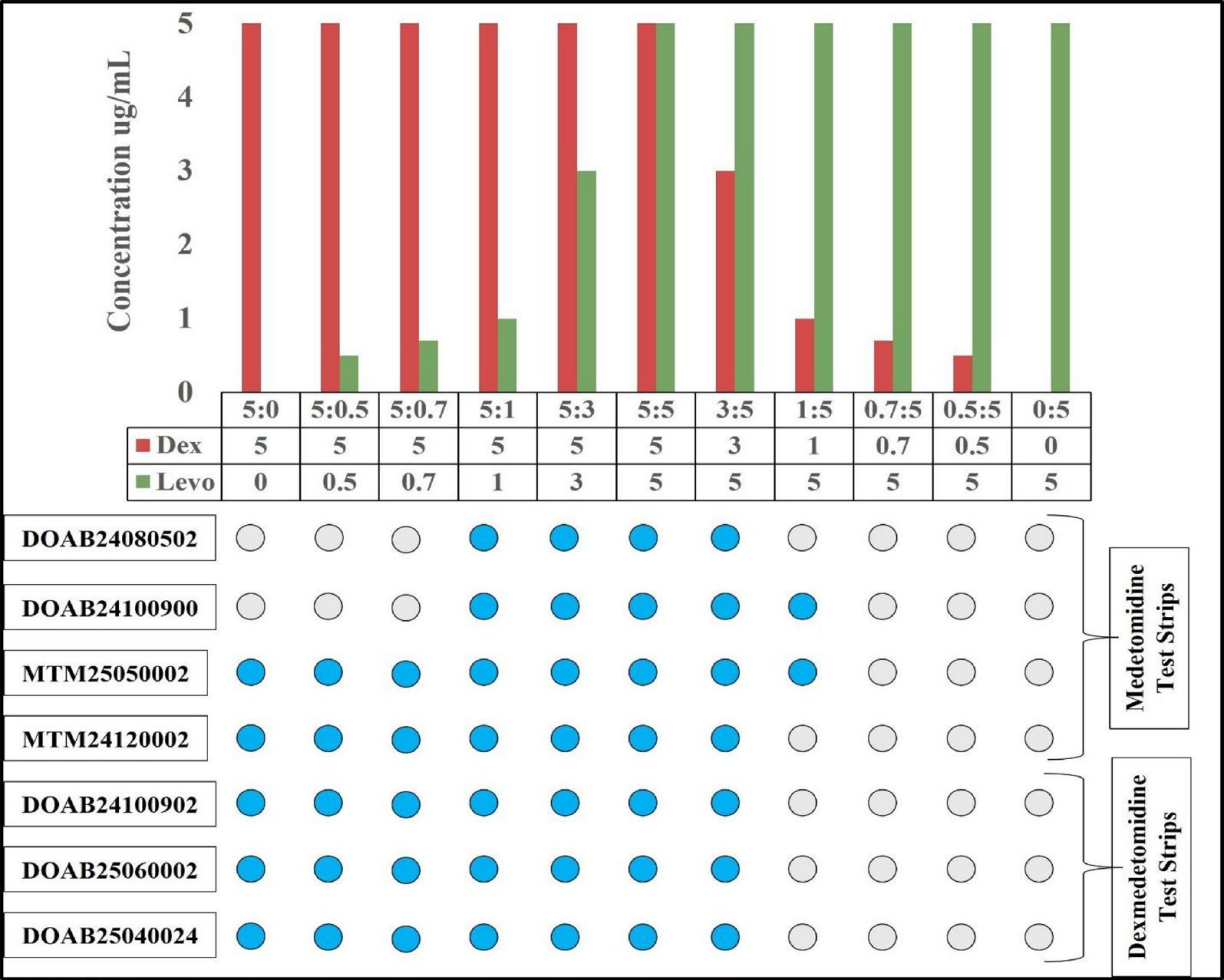
Medetomidine and dexmedetomidine test strips show varying responses to samples that contain different dexmedetomidine and levomedetomidine in different ratios

Racemic medetomidine is a 1:1 mixture of dexmedetomidine and levomedetomidine. There is some ambiguity about how test strips labeled as detecting “medetomidine” might target the drug. The three most recent lots of BTNX medetomidine test strips appear to feature an antibody that is selective for dexmedetomidine; as long as the concentration of this isomer is above the LOD, the strips give positive results (Fig. 4). These strips also respond to racemic medetomidine (at double the LOD level for pure dexmedetomidine, since only the dexmedetomidine in the racemic medetomidine will activate the test strip). This also appears to be the operational mechanism for the Wisebatch medetomidine strips, which gave positive results whenever the concentration of dexmedetomidine (in a mixture of dex and levo) was above 3000 ng/mL for one lot, and 1000 ng/mL for the other, but gave negative results for pure levomedetomidine. However, the two early lots of BTNX strips use a different mechanism. For the lot numbers DOAB24080502 and DOAB24100900, both dex and levo forms of medetomidine were required to be present for these medetomidine strips to read positive. Although the LOD for racemic medetomidine was below 2000 ng/mL for both lots, they yielded negative results when tested with 5000 ng/mL of either pure dexmedetomidine or pure levomedetomidine. This suggests that the test line contains multiple antibodies–eg, one monoclonal antibody against dex and another against levo, or possibly polyclonal antibodies raised against a racemic medetomidine hapten.

### Interference

All lots of medetomidine test strips were assessed for their cross-reactivity with other illicit drugs and common cutting agents. Initially, a 2 mg/mL concentration of each interferant was evaluated. Detomidine HCl caused false positives on all the strips, and levamisole gave faint test lines (which can be misread as false positives) for almost all the strips (Table 2). Xylazine and lidocaine did not interfere at 2 mg/mL concentrations. 

Detomidine is an α₂-adrenergic agonist commonly used as a sedative and analgesic in veterinary medicine, especially in horses. Structurally, it is similar to medetomidine but lacks one methyl group. In their study, da Silva MCC et al. compared dose rates of medetomidine (0.1 mg/kg) and detomidine (0.25 mg/kg) in cats. Medetomidine is about 6.2 times more selective for alpha-2 adrenoceptors over alpha-1 receptors than detomidine [24].

Further testing was conducted on some strips to determine the lowest concentration at which false positive results would be observed for detomidine. For the BTNX lots DOAB24080502, DOAB24100900, and DOAB24100902, even at 0.07 mg/mL, all strips gave positive results. Detomidine ceased interference at 0.02 mg/mL (SI Table S2). Due to the high structural homology of detomidine and medetomidine, it is likely to interfere with many antibodies to medetomidine. Since detomidine is also a veterinary sedative that could be subjected to abuse, this cross-reactivity may be a feature, not a bug (Table 3).

**Table 3:**
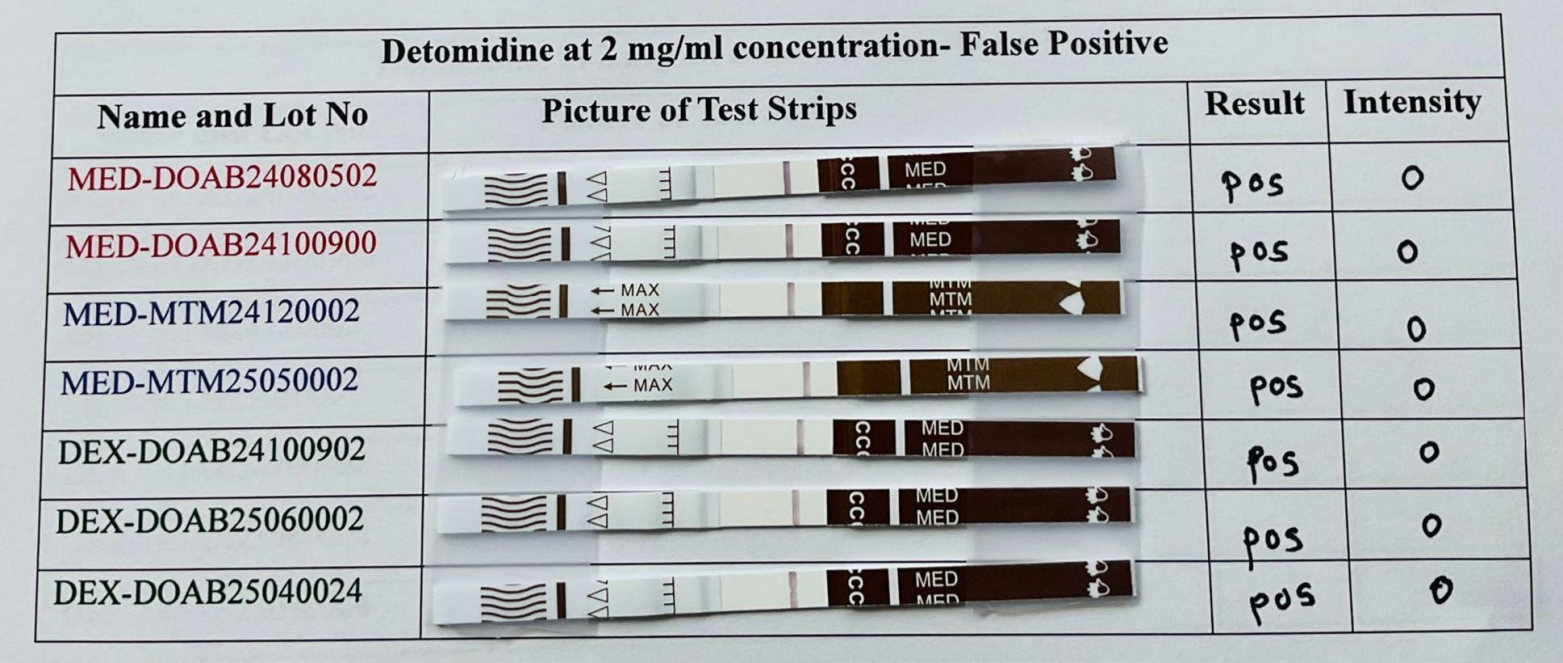
False positive results for detomidine on all lots of medetomidine and dexmedetomidine test strips

## Conclusion

Seven lots of MTS produced by two manufacturers were evaluated under controlled conditions using certified reference materials and laboratory-prepared samples. Based on our observations, all the drug test strips could detect their targets at lower concentrations in soft or deionized water than in hard water, and performance was degraded at temperatures above 37 °C. In communities with very hard water, health organizations that are distributing drug test strips should consider providing 5 mL plastic ampules or vials of purified water along with 10 mg scoops for sample preparation.

One surprising observation in this study is that some medetomidine strips may give a negative result for diverted medical medetomidine (dexmedetomidine) because the strips require both dex and levo to be present to give a positive result. We note that the manufacturer whose initial lots of products could not detect dexmedetomidine when it was the only form of the drug present in the sample has since reformulated their test strip, which now can detect both racemic medetomidine and dexmedetomidine. This chiral sensitivity profile is more suitable for harm reduction applications where the main concern is detection of sedative activity from medetomidine. However, there could be a forensic application for strips that require both dex and levo for a positive signal: if a sample is positive on a strip that detects only dexmedetomidine, but negative on the racemic medetomidine detector strip, it could help identify homochiral dexmedetomidine, hence possible diversion of human medical medetomidine. Determination of the chiral composition of seized drug material is most commonly done for methamphetamine using GC–MS or LC–MS [25]. Sisco et al. evaluated illicit medetomidine forms in the samples that were obtained through the Rapid Drug Analysis and Research (RaDAR) program [26]. In total, 100 medetomidine-positive items were analyzed, consisting of 12 drug product samples and 88 residue samples from drug paraphernalia. These materials were collected from 12 different sites across five East Coast states between August 2024 and February 2025. Chiral LC–MS results suggest that most of the medetomidine in the illicit drug supply is currently racemic material [26].

Users of MTS should exercise caution when extrapolating these findings to other MTS brands or lots. We observed both brand-to-brand and lot-to-lot variation in the performance of medetomidine test strips. These differences result from different test strip designs, which may include use of different assay designs (eg, for a noncompetitive assay, the targeting antibody could be incorporated on the test line and the hapten on the conjugate nanoparticles, or vice versa) and use of different monoclonal or polyclonal antibodies with different selection and cutoff criteria, leading to variations in sensitivity and specificity across different lot numbers. The availability of all these different MTS designs and the lack of a uniform labeling convention to indicate whether a manufacturer’s antibody has changed from one lot to another makes it challenging for health authorities and community-based organizations to determine the most suitable MTS to distribute for public health purposes.

## Supplementary Information

Below is the link to the electronic supplementary material.

Supplementary Material 1.

## Data Availability

Data is provided within the manuscript or supplementary information files.
