# Supplementary material for: Chiral sensitivity of medetomidine lateral flow immunoassay test strips
Source: Harm Reduct J. 2026 Jan 2;23:19. doi: 10.1186/s12954-025-01387-6 (PMC12866554; doi:10.1186/s12954-025-01387-6)
Supplement: Supplementary file 1 — Supplementary Material 1. [file 12954_2025_1387_MOESM1_ESM.docx]

# Supporting information

Chiral Sensitivity of Medetomidine Lateral Flow Immunoassay Test Strips

Anita Amate, Marya Lieberman

Supporting information 1

Fig. S1a Photos of strips that have been run with water (true negative) 2

Fig. S1b Photos of strip packaging 3

Table S1. Quantitative LOD data for medetomidine and dexmedetomidine 4

Fig. S2 Chemical structures of compounds tested for interference 4

Table S2. False positive results for detomidine on medetomidine and dexmedetomidine test strips 5

Table S3. Medetomidine test strips result for BTNX Med-900 and Med-502 varying dex: levo enantiomer ratios 6

Table S4. Medetomidine test strips result for WiseBatch Med-20002 and Med-50002, varying dex: levo enantiomer ratios 7

## Fig. S1a Photos of strips that have been run with water (true negative)


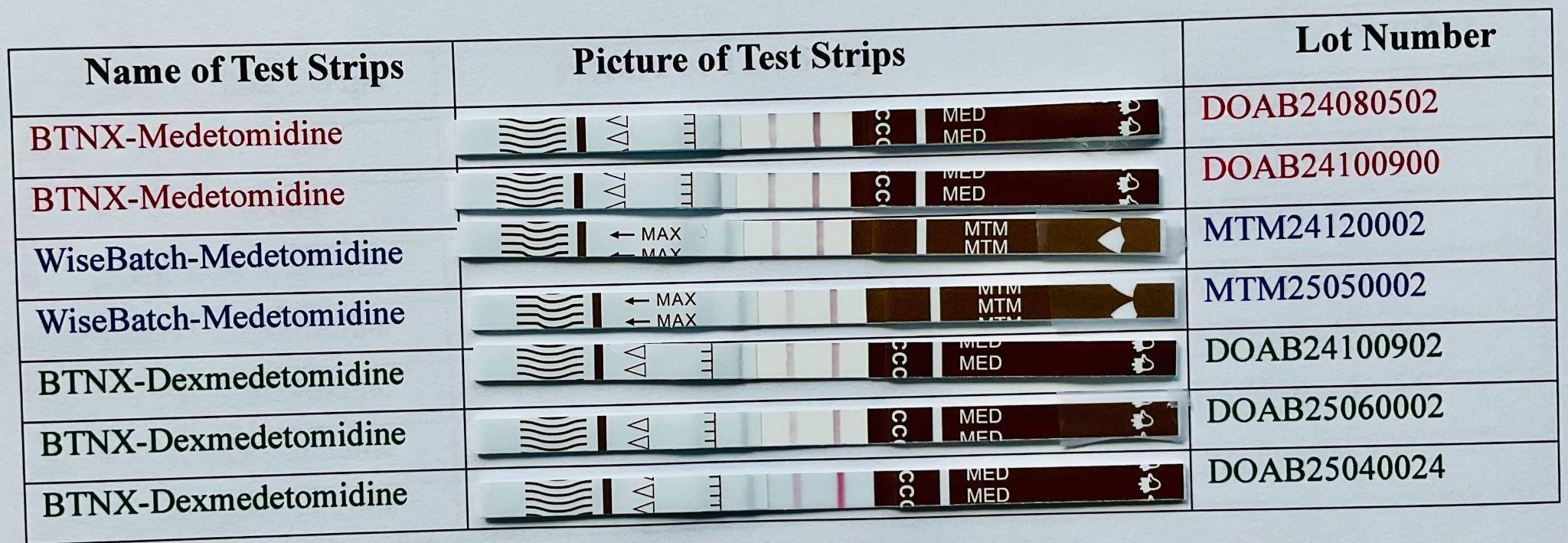


Photographs of the lateral flow immunoassay strips tested in this study.  The brand and lot number corresponding to each strip are shown to the left and right of the strip.

## Fig. S1b Photos of strip packaging


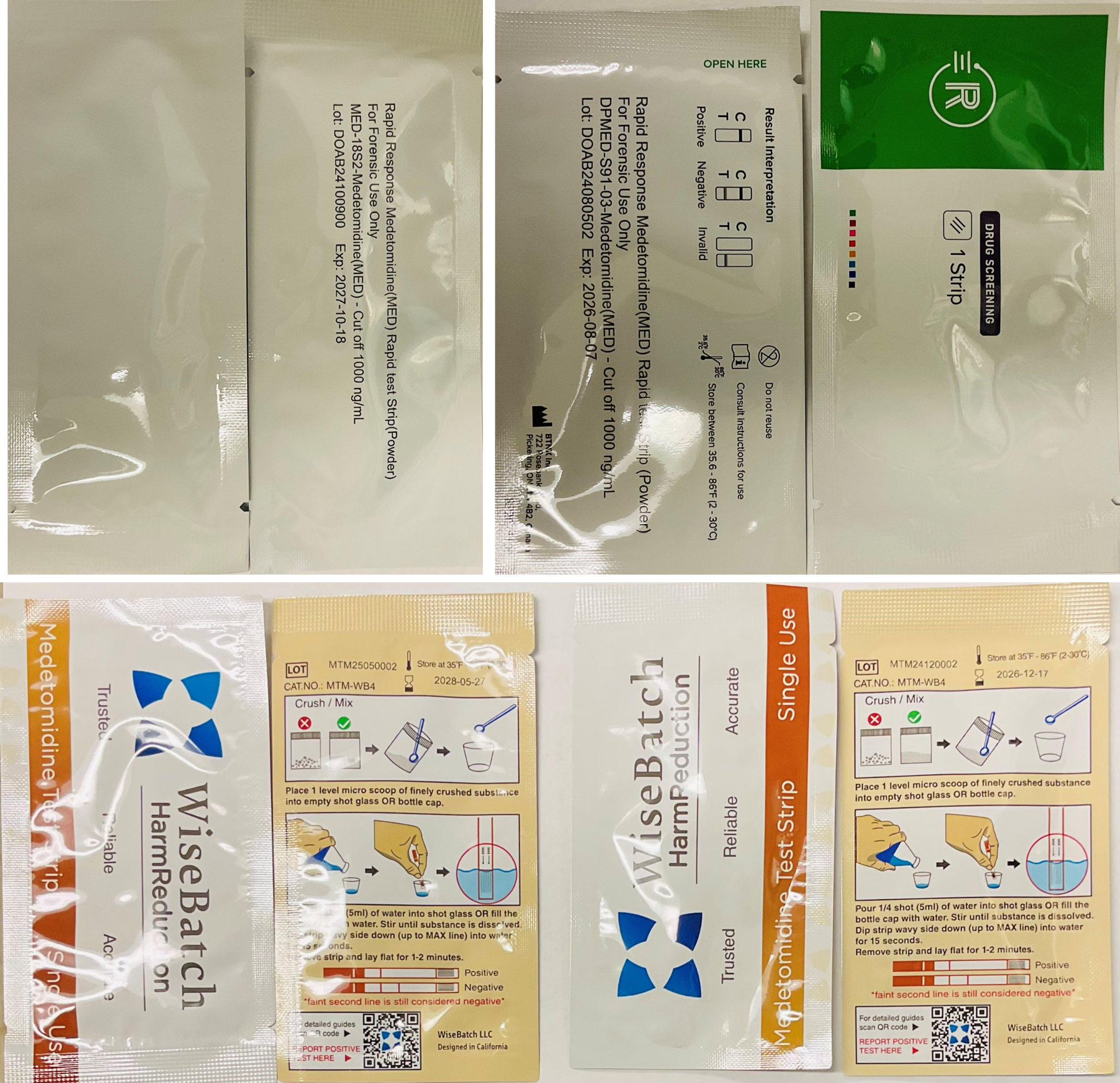


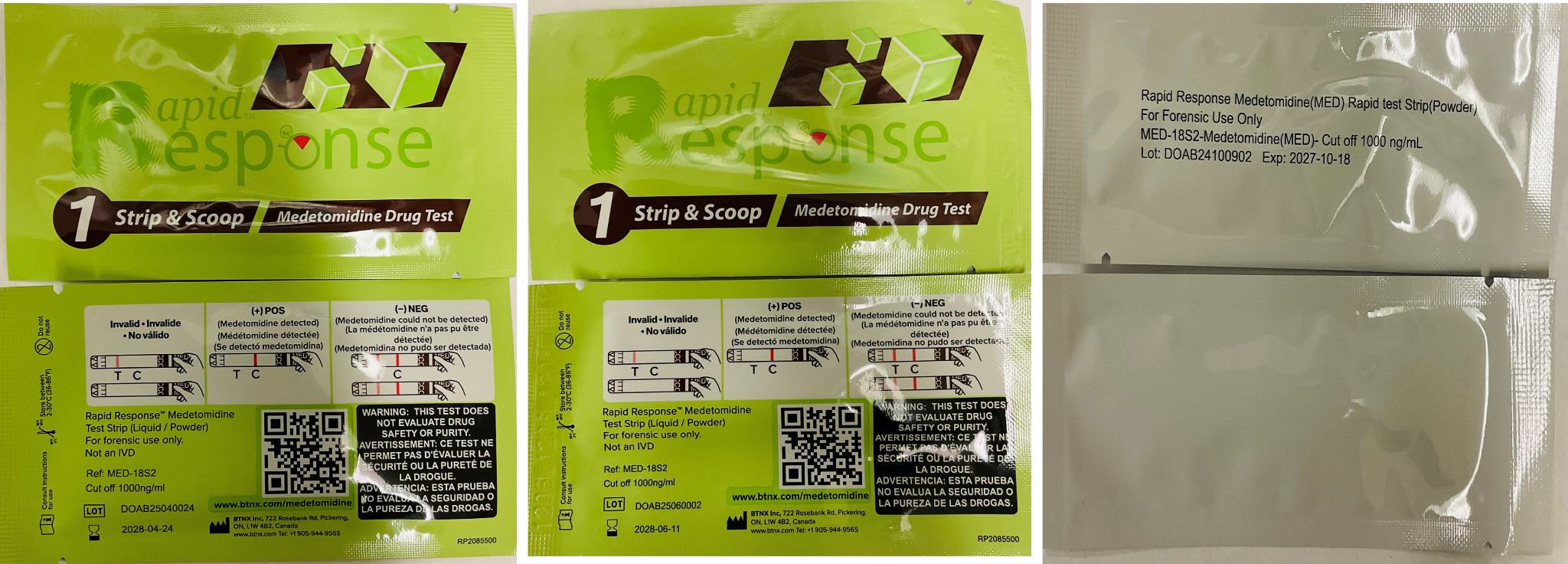


## Table S1. Quantitative LOD data for medetomidine and dexmedetomidine

| Brand | Lot Number | DI Water | Tap Water | At 38°C | At 5°C |
| --- | --- | --- | --- | --- | --- |
| BTNX-Med | DOAB24080502 | 1200 ng/mL | 1500 ng/mL | 1800 ng/mL | 1000 ng/mL |
| BTNX-Med | DOAB24100900 | 2000 ng/mL | 2500 ng/mL | 1200 ng/mL | 1200 ng/mL |
| WiseBatch-Med | MTM25050002 | 700 ng/mL | 700 ng/mL | 1000 ng/mL | 1000 ng/mL |
| WiseBatch-Med | MTM24120002 | 1000 ng/mL | 1200 ng/mL | 2500 ng/mL | 900 ng/mL |
| BTNX-Dex | DOAB24100902 | 900 ng/mL | 1500 ng/mL | 2500 ng/mL | 1500 ng/mL |
| BTNX-Dex | DOAB25060002 | 500 ng/mL | 500 ng/mL | 500 ng/mL | 700 ng/mL |
| BTNX-Dex | DOAB25040024 | 1500 ng/mL | 1500 ng/mL | 1500 ng/mL | 1200 ng/mL |

## Fig. S2 Chemical structures of compounds tested for interference


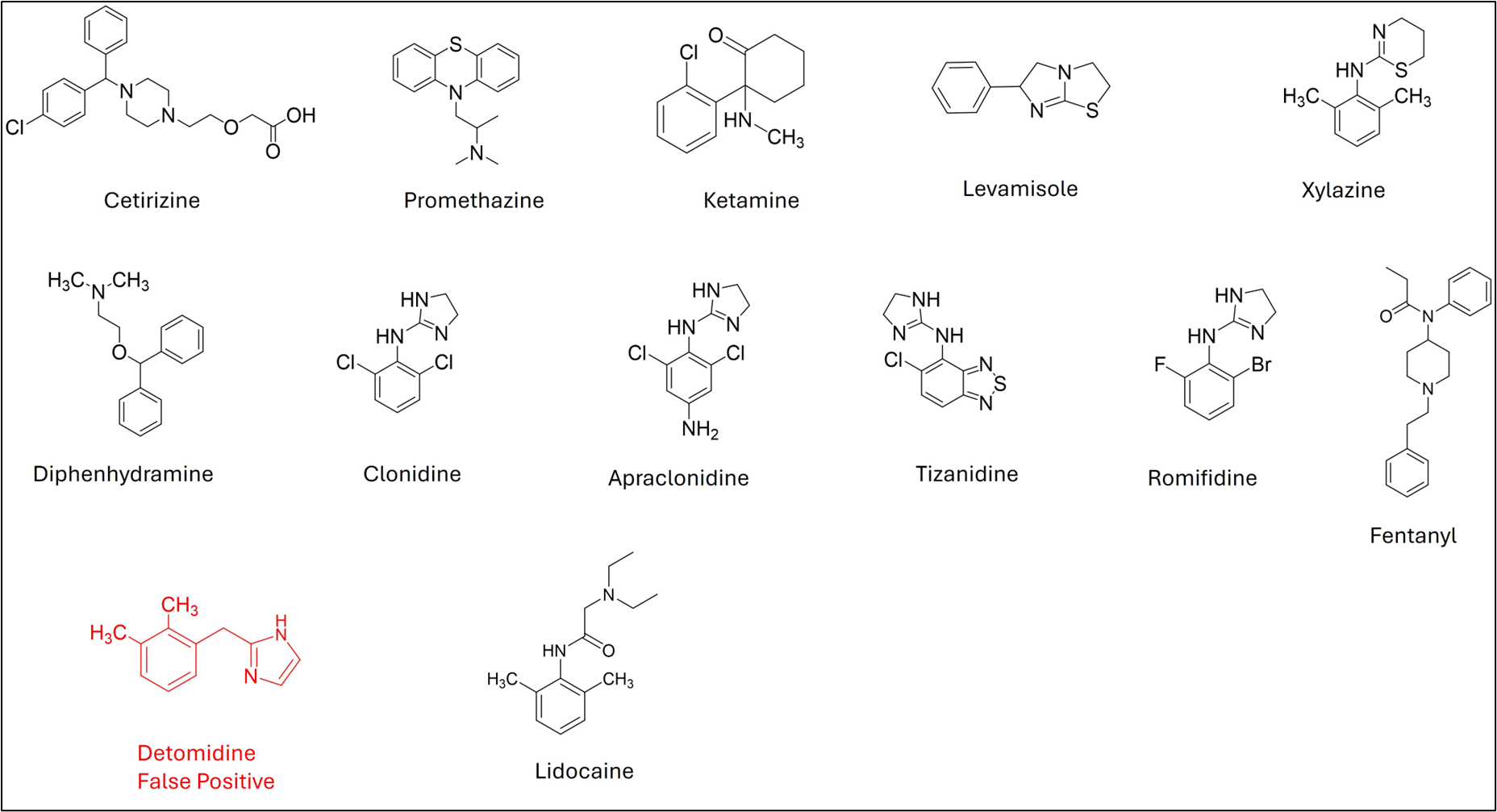


## Table S2. False positive results for detomidine on medetomidine and dexmedetomidine test strips

| Conc | Type of test strips | Result | Intensity | Picture |
| --- | --- | --- | --- | --- |
| 2 mg/ml | Med-502 | Pos | 0 | 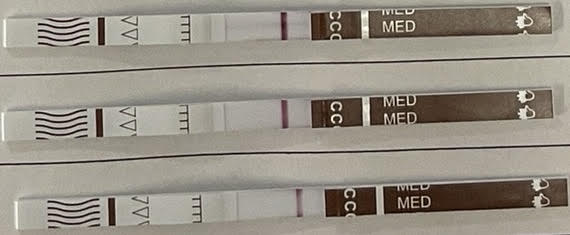 |
|  | Med-900 | Pos | 0 |  |
|  | Dex- 902 | pos | 0 |  |
| 0.7 mg/ml | Med-502 | Pos | 0 | 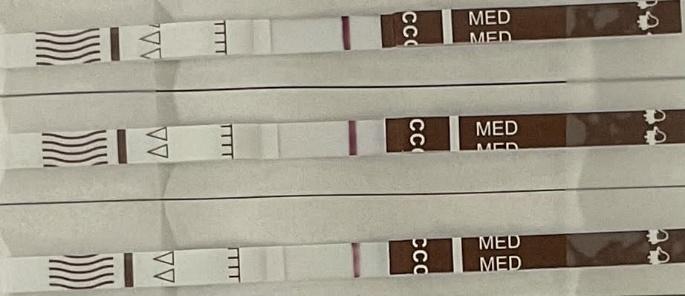 |
|  | Med-900 | Pos | 0 |  |
|  | Dex- 902 | pos | 0 |  |
| 0.2 mg/ml | Med-502 | Pos | 0 | 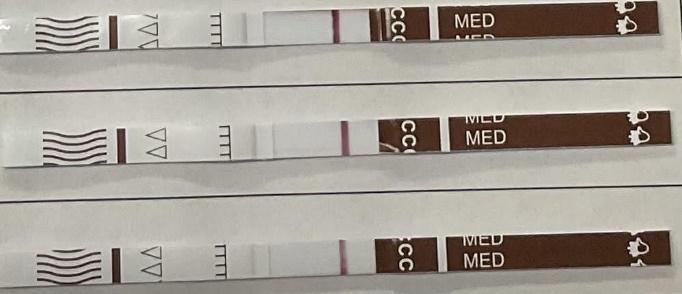 |
|  | Med-900 | Pos | 0 |  |
|  | Dex- 902 | pos | 0 |  |
| 0.07 mg/ml | Med-502 | Pos | 0 | 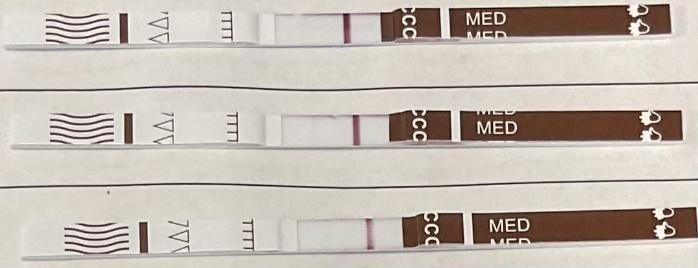 |
|  | Med-900 | Pos | 0 |  |
|  | Dex- 902 | pos | 0 |  |
| 0.02 mg/ml | Med-502 | Neg | 4 | 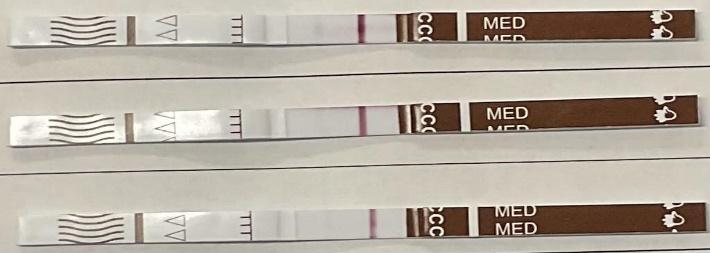 |
|  | Med-900 | Neg | 3 |  |
|  | Dex- 902 | Neg | 2 |  |

##

## Table S3. Medetomidine test strips result for BTNX Med-900 and Med-502 varying dex: levo enantiomer ratios

| Conc. Dex: Levo | Test Strips Photo  Lot- Med DOAB24100900 | Result | Intensity |
| --- | --- | --- | --- |
| 5: 3 ug/mL | 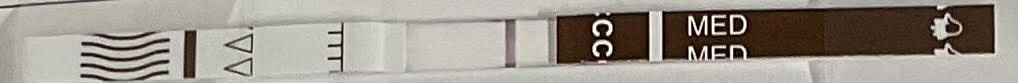 | Positive | 0 |
| 5: 1 ug/mL | 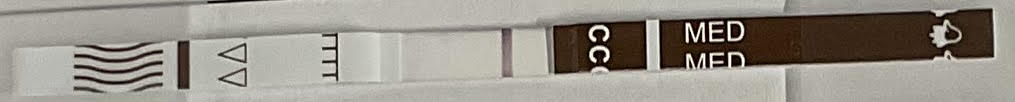 | Positive | 0 |
| 5: 0.7 ug/ml | 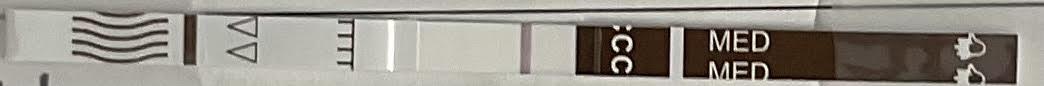 | Negative | 1 |
| 5: 0.5 μg/mL | 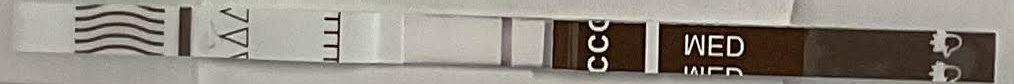 | Negative | 3 |
|  | | | |
| Conc. Levo: Dex | Lot- Med DOAB24100900 | Result | Intensity |
| 5: 3 ug/mL | 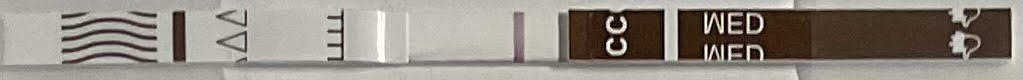 | Positive | 0 |
| 5: 1 ug/mL | 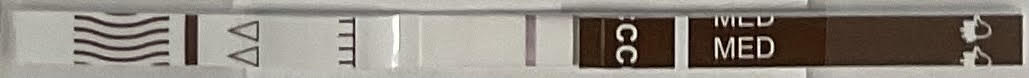 | Negative | 2 |
| 5: 0.7 ug/ml | 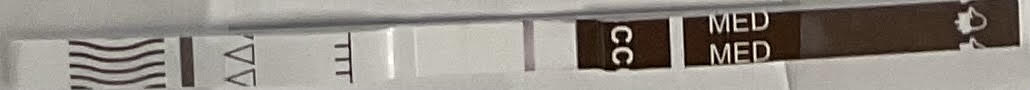 | Negative | 3 |
| 5: 0.5 ug/mL | 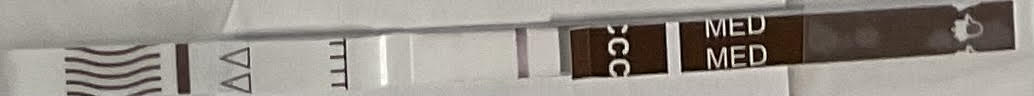 | Negative | 3 |
|  | | | |
| Conc. Dex: Levo | Lot- Med DOAB24080502 | Result | Intensity |
| 5: 3 ug/mL | 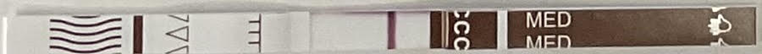 | Positive | 0 |
| 5: 1 ug/mL | 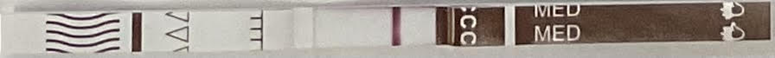 | Positive | 0 |
| 5: 0.7 ug/ml | 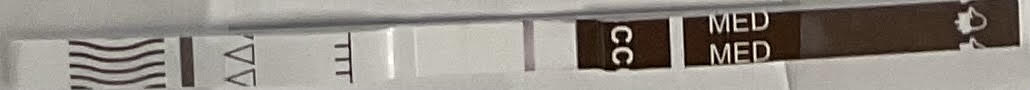 | Negative | 1 |
| 5: 0.5 ug/mL | 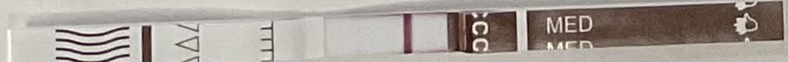 | Negative | 3 |
|  | | | |
| Conc. Levo: Dex | Lot- Med DOAB24080502 | Result | Intensity |
| 5: 3 ug/mL | 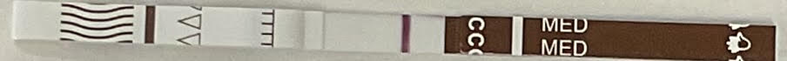 | Positive | 0 |
| 5: 1 ug/mL | 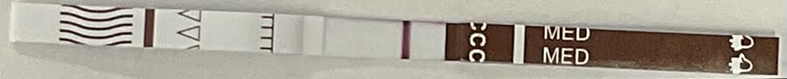 | Positive | 0 |
| 5: 0.7 ug/ml | 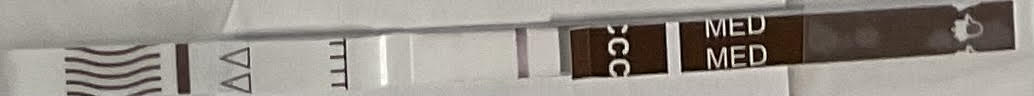 | Negative | 3 |
| 5: 0.5 ug/mL | 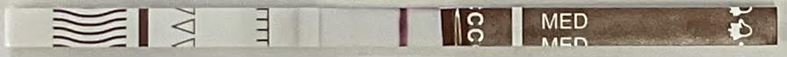 | Negative | 3 |

##

## Table S4. Medetomidine test strips result for WiseBatch Med-20002 and Med-50002, varying dex: levo enantiomer ratios

| Conc. Dex: Levo | Test Strips Photo  Lot- Med MTM24120002 | Result | Intensity |
| --- | --- | --- | --- |
| 5: 3 ug/mL | 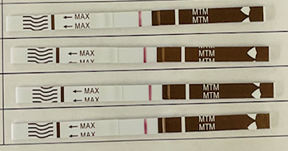 | Positive | 0 |
| 5: 1 ug/mL |  | Positive | 0 |
| 5: 0.7 ug/ml |  | Positive | 0 |
| 5: 0.5 ug/mL |  | Positive | 0 |
|  | | | |
| Conc. Levo: Dex | Lot- Med MTM24120002 | Result | Intensity |
| 5: 3 ug/mL | 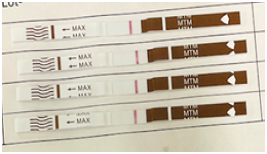 | Positive | 0 |
| 5: 1 ug/mL |  | Negative | 2 |
| 5: 0.7 ug/ml |  | Negative | 4 |
| 5: 0.5 ug/mL |  | Negative | 3 |
|  | | | |
| Conc. Dex: Levo | Lot- Med MTM25050002 | Result | Intensity |
| 5: 3 ug/mL | 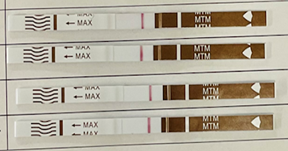 | Positive | 0 |
| 5: 1 ug/mL |  | Positive | 0 |
| 5: 0.7 ug/ml |  | Positive | 0 |
| 5: 0.5 ug/mL |  | Positive | 0 |
|  | | | |
| Conc. Levo: Dex | Lot- Med MTM25050002 | Result | Intensity |
| 5: 3 ug/mL | 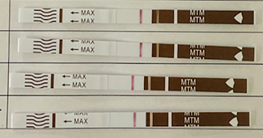 | Positive | 0 |
| 5: 1 ug/mL |  | Positive | 0 |
| 5: 0.7 ug/ml |  | Negative | 3 |
| 5: 0.5 ug/mL |  | Negative | 4 |

**Medetomidine BSA Conjugate:- AAT Bioquest company product (**[**https://www.aatbio.com**](https://www.aatbio.com/)**)**

The medetomidine-BSA conjugate is a synthetic immunogen developed to generate antibodies against medetomidine. Due to its small molecular size and poor inherent immunogenicity, medetomidine is chemically conjugated to a carrier protein, bovine serum albumin (BSA), using a defined linker to ensure stable attachment while maintaining the drug’s key antigenic features. This conjugate is suitable for use in antibody production and immunoassay development, including ELISA, competitive binding assays, and lateral flow tests, for the detection of medetomidine or related compounds.


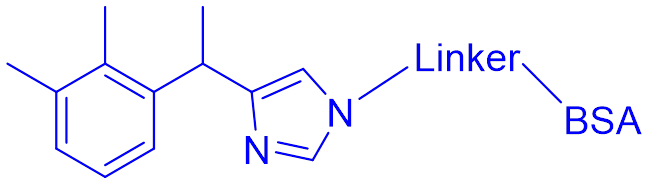


**Figure S3.** Medetomidine BSA Conjugate
